# Screening and identification of key candidate genes and pathways in myelodysplastic syndrome by bioinformatic analysis

**DOI:** 10.7717/peerj.8162

**Published:** 2019-11-29

**Authors:** Ying Le

**Affiliations:** Department of Hematology, Maoming People’s Hospital, Maoming, Guangdong, China

**Keywords:** Myelodysplastic syndrome, Diagnosis, GEO, Molecular mechanism, Survival analysis, Prognosis

## Abstract

Myelodysplastic syndrome (MDS) is a heterogeneous hematologic malignancy derived from hematopoietic stem cells and the molecular mechanism of MDS remains unclear. This study aimed to elucidate potential markers of diagnosis and prognosis of MDS. The gene expression profiles GSE19429 and GSE58831 were obtained and downloaded from the Gene Expression Omnibus (GEO) database. The differentially expressed genes (DEGs) in MDS were screened using GEO2R and overlapped DEGs were obtained with Venn Diagrams. Then, Gene Ontology and Kyoto Encyclopedia of Genes and Genomes pathway functional enrichment analyses, protein–protein interaction network establishment and survival analyses were performed. Functional enrichment analysis indicated that these DEGs were significantly enriched in the interferon signaling pathway, immune response, hematopoietic cell lineage and the FOXO signaling pathway. Four hub genes and four significant modules including 25 module genes were obtained via Cytoscape MCODE. Survival analysis showed that the overall survival of MDS patients having BLNK, IRF4, IFITM1, IFIT1, ISG20, IFI44L alterations were worse than that without alterations. In conclusion, the identification of these genes and pathways helps understand the underlying molecular mechanisms of MDS and provides candidate targets for the diagnosis and prognosis of MDS.

## Introduction

Myelodysplastic syndrome (MDS) is a clonal hematopoietic stem cell (HSC) disease, mainly involving cytogenetic changes and/or genetic mutations, and there is also a widespread gene hypermethylation at advanced stage (Adès, Itzykson & Fenaux, 2014). The main features of MDS are myeloid cell cytopenias, morphologic dysplasia, ineffective hematopoiesis and a high risk of transformation to acute myeloid leukemia (AML) (Weinberg & Hasserjian, 2019). Although there are many new drugs for the treatment of MDS in recent years, about one-third of patients with MDS experience transformation to AML and the overall survival (OS) of MDS remains not ideal (Duchmann & Itzykson, 2019). There is accumulating evidence that MDS are associated with abnormal genetic mutations (Ogawa, 2019). Therefore, identifying more molecular biomarkers is critical for early diagnosis, treatment and prognosis of MDS.

In recent years, gene chip technology has developed rapidly and has been widely used in gene detection (Vogelstein et al., 2013). It has been confirmed that the pathogenesis and progression and heterogeneity of MDS are closely related to the genetic landscape (Maciejewski & Balasubramanian, 2017), such as tet methylcytosine dioxygenase 2 (TET2), isocitrate dehydrogenase1/2 (IDH1/2), additional sex comb-like 1 (ASXL1), enhancer of zeste homolog 2 (EZH2), NRAS proto-oncogene (NRAS), RUNX family transcription factor 1 (RUNX1) and GATA binding protein 2 (GATA2). TET2 is the most common mutations in MDS and it is reported that TET2 deficiency disturbed erythroid differentiation, contributing to ineffective erythropoiesis (Qu et al., 2018). IDH1/2 mutation was positively correlated with bone marrow blast counts and negatively correlated with absolute neutrophil counts. Patients with IDH1 mutations had shorter OS and progression-free survival (PFS), while IDH2 mutations had no effect on OS or PFS of MDS patients (Wang et al., 2017). The ASXL1 mutation can significantly and independently predict adverse outcomes of myeloid tumors (Mangaonkar et al., 2018). Aberrant EZH2 splicing and/or EZH2 mutations which impaired histone H3 lysine 27 tri-methylation may be associated with the pathogenesis of MDS (Shirahata-Adachi et al., 2017). NRAS was thought to be significantly associated with MDS, especially in the progression to leukemia (Leite et al., 2017; Makishima, 2019). RUNX1 mutation was observed in approximately 10% of patients with MDS. Both the repressor and activator of RUNX1 resulted in the tumor suppressor activity in MDS and AML (Behrens et al., 2016). GATA2 has been shown to be related to the progression of MDS, such as the progression from low-risk to high-risk or AML (Makishima, 2019; McReynolds et al., 2019). Although the discovery of these genes contributes to the research of MDS, the pathogenesis of MDS remains unclear. Re-integration and distribution of these abnormal genes provides the possibility to identify diseases associated with biological markers. The bioinformatics approach provides a good platform to correlate gene expression profiles with disease.

In this study, I downloaded two microarray datasets from the Gene Expression Omnibus (GEO) database to obtain differentially expressed genes (DEGs) from bone marrow CD34+ cells of MDS and healthy controls. Then, I performed Gene Ontology (GO), Kyoto Encyclopedia of Genes and Genomes (KEGG) and protein–protein interaction (PPI) network analyses on these DEGs. Finally, I screened the module genes and the hub genes and analyzed the relationship between gene expression and prognosis to help understand the underlying molecular mechanism of MDS.

## Materials and Methods

### Microarray data information

GEO (http://www.ncbi.nlm.nih.gov/geo) is a public genomics data repository created and maintained by the National Center for Biotechnology Information (NCBI), which contains high-throughput gene expression data, chips and microarrays. The gene expression profiles GSE19429 and GSE58831 of MDS were obtained and downloaded from GEO. These two gene expression datasets are based on the GPL570 platform (HG-U133_Plus_2 Affymetrix Human Genome U133 Plus 2.0 Array). GSE19429 dataset contained 183 MDS patients and 17 healthy controls. GSE58831 dataset contained 159 MDS patients and 17 healthy controls. Bone marrow samples were obtained and CD34+ cells isolated from MDS patients and healthy controls.

### Identification of DEGs and data processing

GEO2R (https://www.ncbi.nlm.nih.gov/geo/geo2r/) is an interactive web tool that can compare two or more sets of samples from the GEO series to identify different genes under different experimental conditions. The DEGs between MDS patients and healthy controls samples were screened using GEO2R. Probe sets without corresponding gene symbols were removed. In this study, *P*-value < 0.05 and (logFC (fold change)) ≥ 1 were considered statistically significant. I also used the online tool Venn diagrams (http://bioinformatics.psb.ugent.be/webtools/Venn/) to identify the overlapped DEGs in the GSE19429 and GSE58831 datasets.

### GO and KEGG pathway enrichment analyses

DAVID (http://david.ncifcrf.gov) is a bioinformatics database that integrates biological data and analysis tools to provide systematic, comprehensive biological functional annotation information for large-scale genes or proteins. I used DAVID to perform a functional enrichment analysis, which included GO and KEGG pathway analyses. GO term enrichment analysis includes molecular function (MF), cellular component (CC) and biological process (BP). KEGG is a database resource that combine of genomic information and higher-level functional information by computerizing processing of known BPs in cells and systematically analyzing the existing gene functions. Pathway analysis can graphically show intracellular BPs such as metabolism, membrane transporting, signaling and cell growth cycles. *P*-value < 0.05 was considered statistically significant.

### PPI network establishment and module analysis

Search Tool for the Retrieval of Interacting Genes (STRING, http://string-db.org) is an open, free online system that provides predicted and experimental interactions of genes and proteins. I used STRING online database to predict and construct the PPI network. In the present study, STRING was used to construct PPI network of DEGs for MDS and the confidence score of the interaction >0.4 was considered statistically significant. Cytoscape is a public software that can graphically display the network and analyze and edit. In this study, I used cytoscape to annotate and beautify the up-downregulated genes of PPI. Molecular Complex Detection (MCODE) is an APP of a plug-in Cytoscape to find densely connected regions for clustering a given network. The PPI networks were mapped using Cytoscape and the significant modules in the PPI networks were identified using MCODE. The screening criteria for the module genes were as follows: degree cutoff = 2, node score cutoff = 0.2, *k*-score = 2 and Max depth = 100. Subsequently, I used STRING to perform GO and KEGG pathway enrichment analyses on the module genes.

### Hub genes selection and co-expression analysis

The screening of hub genes is mainly carried out through the MCODE plug-in of cytoscape. CBioPortal (http://www.cbioportal.org) is an online analysis platform for multidimensional cancer genomic data that visualizes genes, samples and data types. In this study, I constructed a network of hub genes and their co-expression genes via cBioPortal. Then I used the BiNGO of Cytoscape plug-in to perform BP analysis and visualization of the hub genes. Finally, I performed hierarchical clustering of the hub genes through UCSC Cancer Genomics Browser (http://genome-cancer.ucsc.edu).

### Survival analysis

The OS analysis of hub genes and module genes were performed using Kaplan–Meier curve in cBioPortal. *P*-value < 0.05 was considered statistically significant.

## Results

### Identification of DEGs in MDS

**Figure 1:**
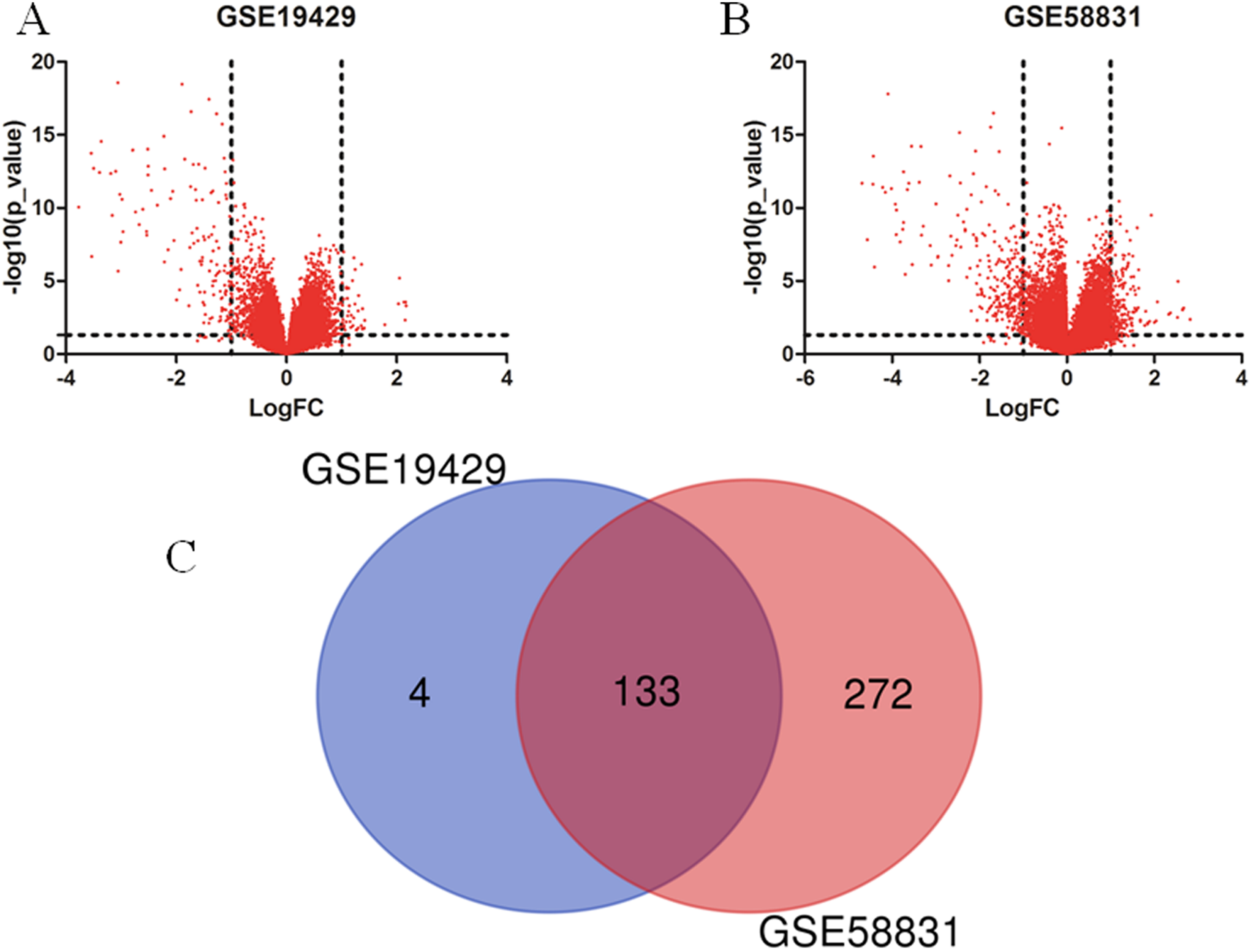
Identification of DEGs in two datasets (GSE19429 and GSE58831). (A) Volcano plot of GSE19429. (B) Volcano plot of GSE58831. (C) Venn diagram. DEGs were selected with (LogFC) ≥ 1 and *P* < 0.05 among the two sets (GSE19429 and GSE58831).

I obtained gene expression profiles from the GSE19429 to GSE58831 datasets of CD34+ cells of bone marrow samples from MDS patients and healthy controls and analyzed DEGs using GEO2R. As shown in Figs. 1A and 1B, I identified 179 and 500 DEGs from GSE19429 to GSE58831, respectively. The overlap among the two datasets between MDS patients and healthy control contained 133 genes as shown in the Venn diagram (Fig. 1C).

### GO and KEGG pathway enrichment analyses of DEGs

**Figure 2:**
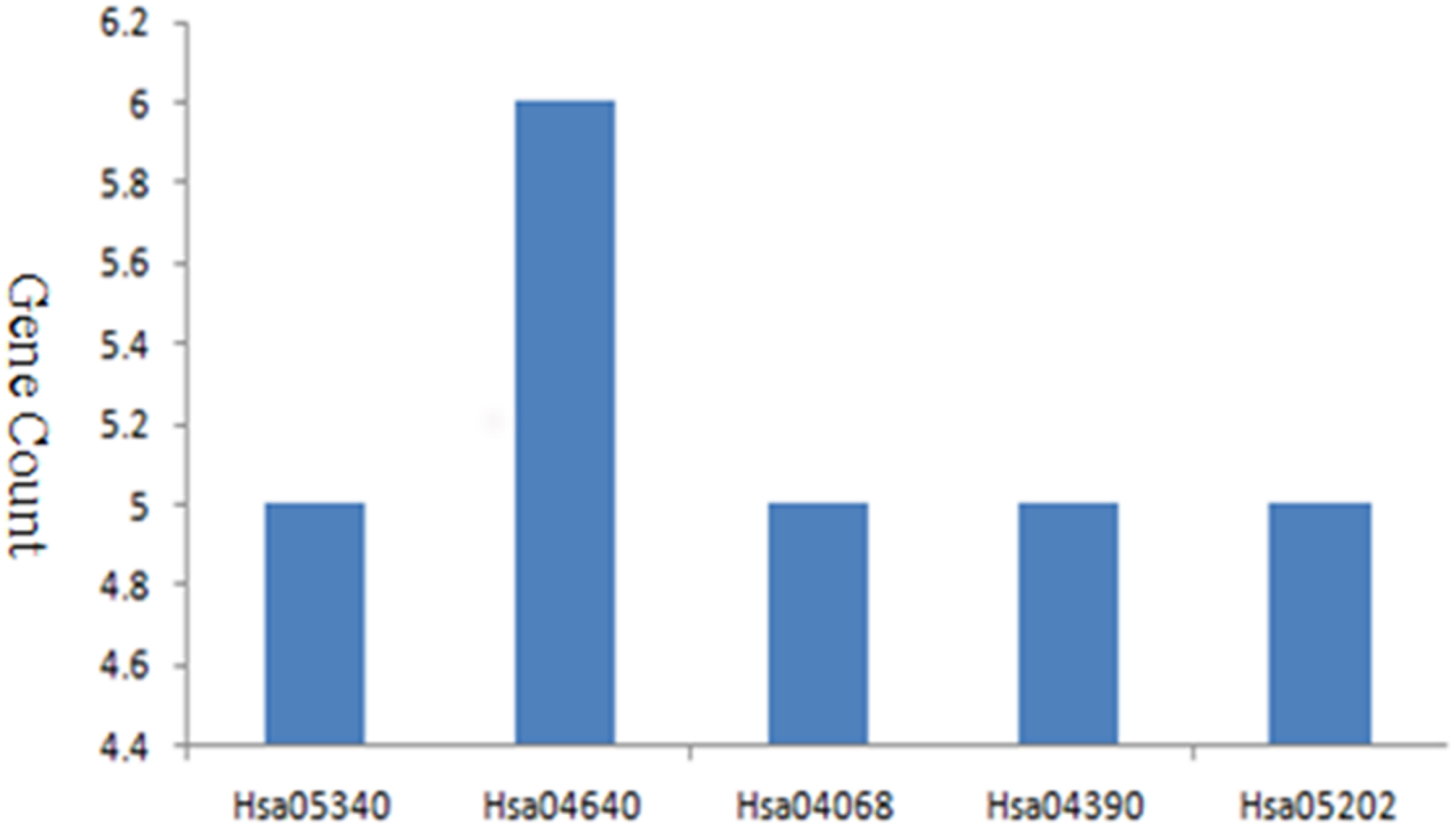
KEGG pathway enrichment analysis of DEGs in MDS.

I used DAVID to perform and analyze the biological classification of DEGs, which included GO analysis and KEGG pathway enrichment analysis. GO analysis showed that the function of upregulated genes in DEGs were mainly enriched in type I interferon signaling pathway, defense response, response to external stimulus, immune effector, cell surface receptor signaling pathway, cytokine-mediated signaling pathway, regulation of cell action potential, sensory perception, immune response, Notch signaling pathway (Table 1). Downregulated genes in BP were mainly enriched in immune response, cell surface receptor signaling pathway, cell activation, cell differentiation, regulation of immune system, regulation of macromolecule metabolic, cell-cell adhesion, biosynthetic process, cell proliferation, cell death, specific DNA binding, apoptotic process, regulation of metabolic process, epithelium development, blood vessel morphogenesis, cell migration, muscle tissue development. For CC, downregulated genes were mainly enriched in side of membrane and extracellular space. For MF, enrichment of downregulated genes was primarily in transcription factor activity, receptor binding, signal transducer activity, steroid hormone receptor activity, hormone receptor binding, core promoter binding, macromolecular complex (Table 1). KEGG pathway analysis revealed that enrichment of DEGs was mostly in primary immunodeficiency, hematopoietic cell lineage, FOXO signaling pathway, hippo signaling pathway, transcriptional misregulation in cancer (Fig. 2).

**Table 1:** Gene Ontology term enrichment analysis of DEGs in MDS.

Term	Description	Count	*P*-value
Upregulated			
GO:0060337	Type I interferon signaling pathway	4	2.28E−04
GO:0098542	Defense response	5	4.77E−04
GO:0009605	Response to external stimulus	10	4.04E−03
GO:0002252	Immune effector	6	6.19E−03
GO:0007166	Cell surface receptor signaling pathway	11	6.70E−03
GO:0019221	Cytokine-mediated signaling pathway	5	1.16E−02
GO:0098911	Regulation of cell action potential	2	2.25E−02
GO:0050954	Sensory perception	3	2.69E−02
GO:0006955	Immune response	7	3.50E−02
GO:0045746	Notch signaling pathway	2	4.76E−02
Downregulated			
GO:0006955	Immune response	27	9.26E−09
GO:0007166	Cell surface receptor signaling pathway	33	3.21E−07
GO:0000982	Transcription factor activity	11	4.51E−06
GO:0001775	Cell activation	16	2.84E−05
GO:0040011	Locomotion	20	1.06E−04
GO:0005102	Receptor binding	19	1.14E−04
GO:0045595	Cell differentiation	20	1.20E−04
GO:0002684	Regulation of immune system	15	1.99E−04
GO:0010605	Regulation of macromolecule metabolic	24	5.27E−04
GO:0022407	Cell-cell adhesion	9	5.71E−04
GO:0009891	Biosynthetic process	20	6.39E−04
GO:0004871	Signal transducer activity	19	1.12E−03
GO:0008283	Cell proliferation	20	1.26E−03
GO:0060548	Cell death	13	1.27E−03
GO:0098552	Side of membrane	9	1.54E−03
GO:0043565	Specific DNA binding	14	1.47E−03
GO:0006915	Apoptotic process	19	1.57E−03
GO:0009892	Regulation of metabolic process	24	1.63E−03
GO:0060429	Epithelium development	14	1.72E−03
GO:0048514	Blood vessel morphogenesis	9	2.64E−03
GO:0003707	Steroid hormone receptor activity	4	2.71E−03
GO:0016477	Cell migration	14	4.50E−03
GO:0060537	Muscle tissue development	7	8.93E−03
GO:0005615	Extracellular space	14	2.40E−03
GO:0051427	Hormone receptor binding	4	3.39E−02
GO:0001047	Core promoter binding	4	4.05E−02
GO:0044877	Macromolecular complex	12	4.16E−02

### PPI network establishment and module analysis

**Figure 3:**
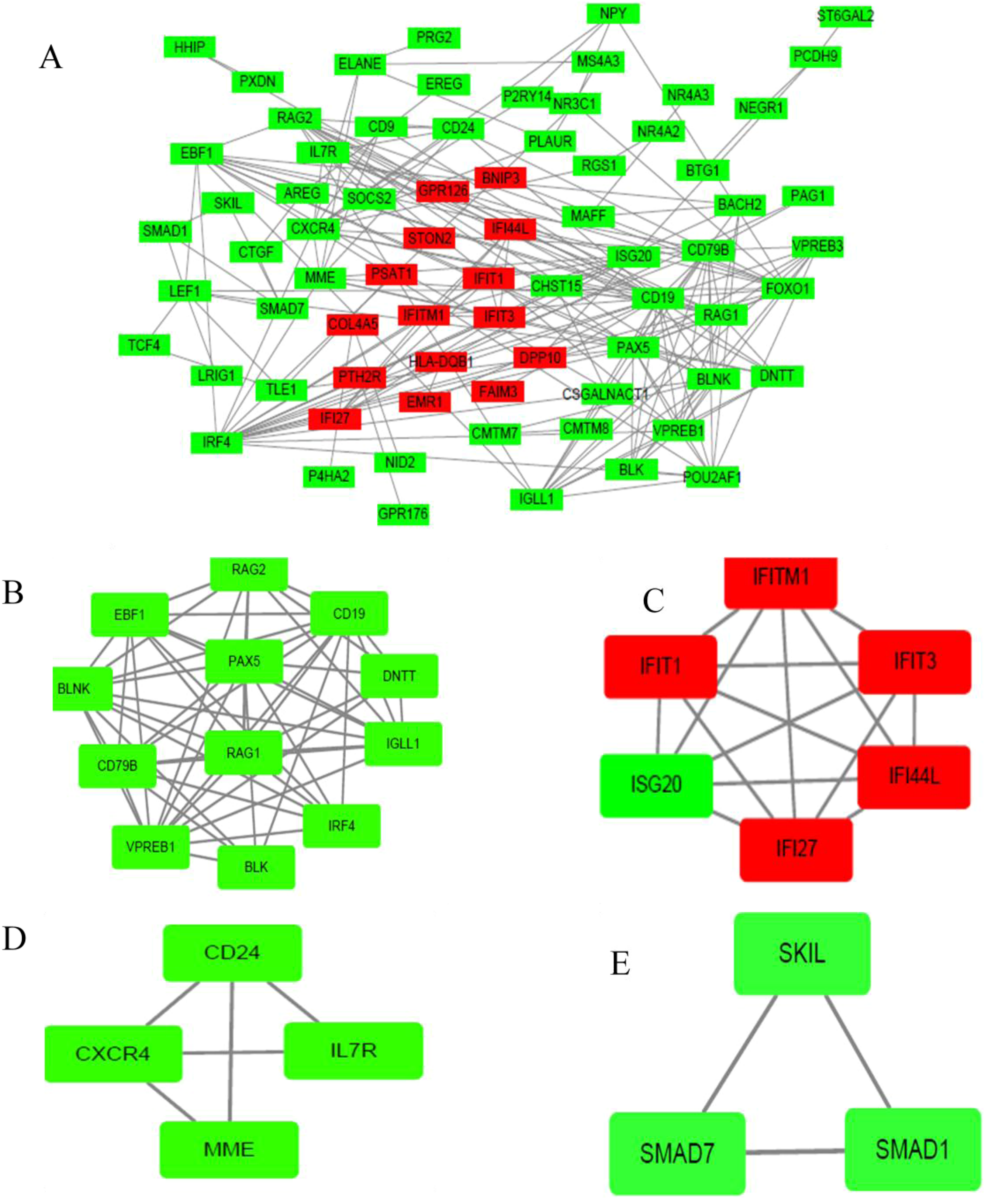
PPI network of DEGs and module analysis. (A) A DEG PPI network was constructed containing 118 DEGs based on the STRING online database (15 upregulated DEGs labeled in red and 103 downregulated DEGs labeled in green). (B) Module analysis based on the degree of importance. Module 1 contains 12 nodes and 53 edges. (C) Module 2 contains six nodes and 15 edges. (D) Module 3 contains four nodes and five edges. (E) Modules 4 contains three nodes and three edges. Upregulated genes are marked in red; downregulated genes are marked in green.

The PPI network of DEGs was established based on the STRING online database and Cytoscape software (15 upregulated and 103 downregulated) (Fig. 3A). There were 133 DEGs in the two datasets, however, 15 intersection genes were not found in the STRING. We obtained four significant modules based on the degree of importance by utilizing cluster analysis of the PPI network in Cytoscape MCODE. Module 1 contained 12 nodes and 53 edges (Fig. 3B); Module 2 contained 6 nodes and 15 edges (Fig. 3C); Module 3 contained 4 nodes and 5 edges (Fig. 3D); Module 4 contained 3 nodes and 3 edges (Fig. 3E). The enrichment analyses of genes involved in these modules were preformed using DAVID. As showed in Table 2, GO analysis indicated these genes in modules were mainly concentrated in immune response, immune cell differentiation, side of membrane, transcription factor, DNA binding, cell adhesion, hemopoiesis, cell activation, defense response, cell surface receptor, DNA metabolic, kidney development. As for KEGG pathway analyses, module genes mainly enriched in primary immunodeficiency, hematopoietic cell lineage, B cell receptor signaling pathway, FOXO signaling pathway.

**Table 2:** Gene Ontology and KEGG pathway enrichment analysis of DEGs in the significant module.

Term	Description	Count	*P*-value
GO:0006955	Immune response	10	3.15E−06
GO:0030098	Immune cell differentiation	6	4.93E−05
GO:0098552	Side of membrane	5	5.71E−04
GO:0000982	Transcription factor	5	4.34E−04
GO:0003677	DNA binding	9	5.97E−04
GO:0016337	Cell adhesion	6	1.24E−03
GO:0030097	Hemopoiesis	6	2.09E−03
GO:0001775	Cell activation	6	2.34E−03
GO:0006952	Defense response	7	2.99E−03
GO:0007166	Cell surface receptor	8	1.22E−02
GO:0006259	DNA metabolic	5	1.83E−02
GO:0001822	Kidney development	3	3.90E−02
Hsa05340	Primary immunodeficiency	5	3.02E−07
Hsa04640	Hematopoietic cell lineage	5	1.36E−05
Hsa04662	B cell receptor signaling pathway	3	6.19E−03
Hsa04068	FOXO signaling pathway	3	2.09E−02

### Hub genes selection and biological analysis

**Figure 4:**
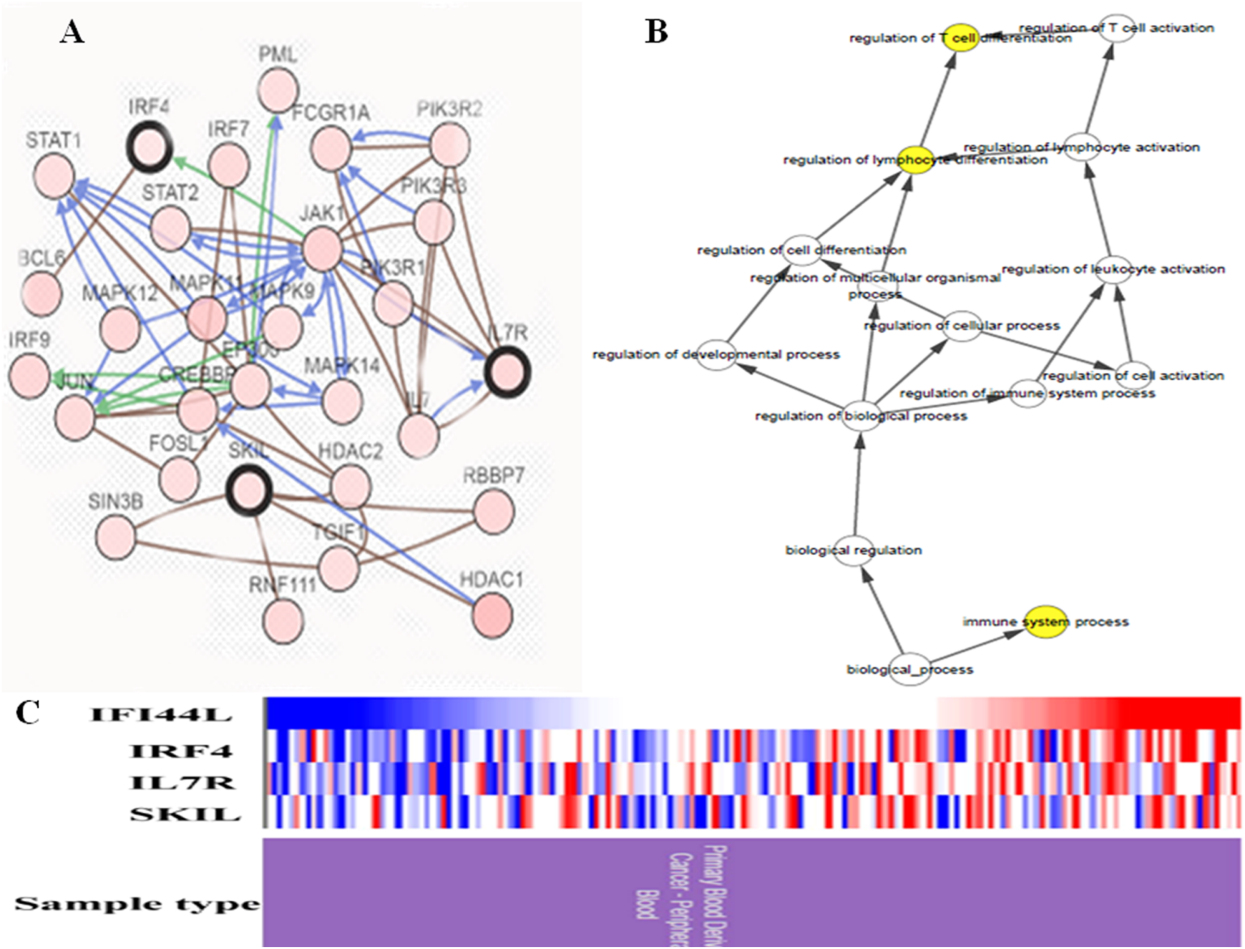
Biological analysis of hub genes. (A) Hub genes and co-expression genes via cBioPortal. (B) The biological process analysis of hub genes via BiNGO. (C) Hierarchical clustering of hub genes via UCSC. (Upregulation of genes are marked in red, downregulation of genes are marked in blue).

In study, a total of four hub genes were identified by MCODE, as IFI44L, IRF4, IL7R, SKIL. I constructed a network of hub genes and their co-expression genes via cBioPortal, but did not find the co-expressed gene of IFI44L in cBioPortal (Fig. 4A). Then I analyzed the BPs of hub genes through BINGO as showed in Fig. 4B. Hierarchical clustering of hub genes was constructed using UCSC and blood derived cancers and healthy controls were roughly identified by hub genes (Fig. 4C).

### Survival analysis

**Figure 5:**
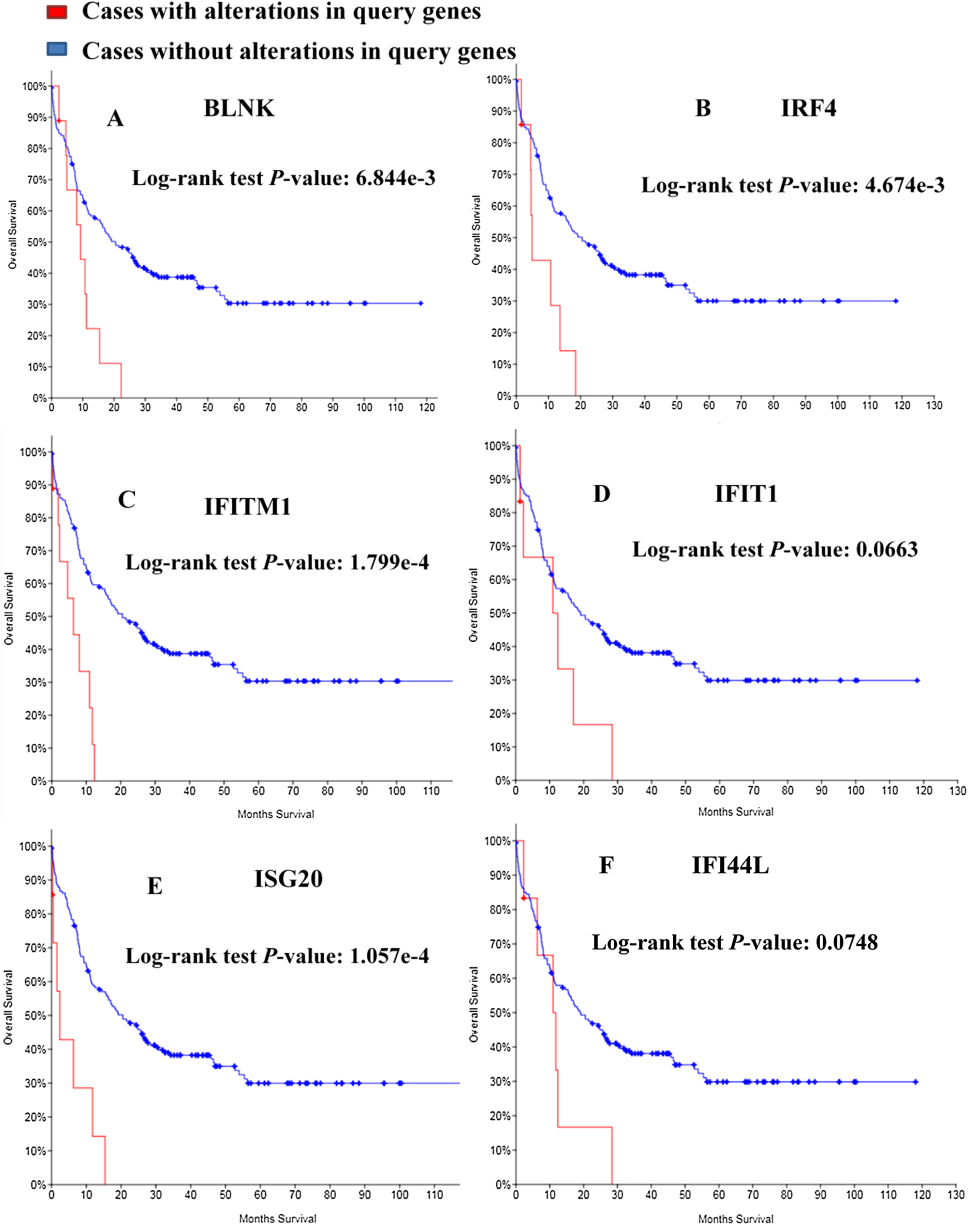
Overall survival analyses of module genes. *P* < 0.05 was considered statistically significant. (A) BLNK; (B) IRF4; (C) IFITM1; (D) IFIT1; (E) ISG20; (F) IFI44L.

Kaplan–Meier curve was employed to predict the prognosis of the 25 identified module genes. First I had a simple understanding of these module genes, such as the full name, abbreviation and function of the genes (Table 3). Among the genes examined, MDS patients having BLNK, IRF4, IFITM1, IFIT1, ISG20, IFI44L alterations showed worse OS (Fig. 5). Although the difference between IFIT1 and IFI44L was not statistically significant, the OS of patients having IFIT1 and IFI44L alteration showed an obvious decline tendency.

**Table 3:** Functional roles of 25 module genes.

No.	Gene symbol	Full name	Function
1	RAG2	Recombination activating 2	RAG2 is involved in B and T cell development and the expression of RAG2 is high in most AMLpatients
2	EBF1	Transcription factor, early B cell factor 1	EBF1 promotes of bone marrow CD34+ cell proliferation and migration,but suppresses the apoptosis of cells
3	BLNK	B cell linker	BLNK regulates the development, maturation and function of B cells
4	CD79B	CD79b molecule	CD79B is necessary for expression and function of the B-cell antigen receptor
5	VPREB1	V-set pre-B cell surrogate light chain 1	VPREB1 is involved in early B cell differentiation and VPREB1 deletion is often detected in relapsed leukemia
6	BLK	BLK proto-oncogene, Src family tyrosine kinase	BLK is involved in cell proliferation and differentiation and has a role in B-cell receptor signaling and B-cell development
7	IRF4	Interferon regulatory factor 4	IRF4 has oncogenic implications in a variety of hematological tumors including multiple myeloma, leukemia and lymphoma
8	IGLL1	Immunoglobulin lambda like polypeptide 1	IGLL1 is involved in cell proliferation and IGLL1 mutation can lead to B cell deficiency and agammaglobulinemia
9	DNTT	DNA nucleotidy-lexotransferase	DNTT acts as a catalyst to add nucleotides during the maturation of B cells and T cells
10	CD19	CD19 molecule	CD19 is a surface-specific marker of B lymphocytes and CD19 decreases the threshold of antigen receptor-dependent
11	PAX5	Paired box 5	PAX5 is involved in cell differentiation and neuro developmentand spermatogenesis
12	RAG1	Recombination activating 1	High expression of RAG1 in B-ALL patients promotes leukemic clonal evolution and high expression of RAG1 is associated with high proliferative markers
13	IFITM1	Interferon induced transmembrane protein 1	IFITM1 promotes proliferation, migration, and invasion of lung cancer
14	IFIT1	Interferon induced protein with tetratricopeptide repeats 1	IFIT1 and IFIT3 promote oral squamous cell carcinoma metastasis and contribute to theanti-tumor effect of gefitinib
15	IFIT3	Interferon induced protein with tetratricopeptide repeats 3	IFIT1 and IFIT3 promote oral squamous cell carcinoma metastasis and contribute to the anti-tumor effect of gefitinib
16	ISG20	Interferon stimulated exonuclease gene 20	ISG20 promotes local tumor immunity and has poor survival in human glioma
17	IFI44L	Interferon induced protein 44 like	High expression of IFI44L is associated with poor prognosis of osteosarcoma
18	IFI27	Interferon alpha inducible protein 27	IFI27 promotes cell proliferation and invasion in oral squamous cell carcinoma
19	CD24	CD24 molecule	CD24 modulates growth and differentiation signals to mature granulocytes and B cells
20	CXCR4	C-X-C motif chemokine receptor 4	High expression of CXCR4 has shorter OS time and shorter relapse-free survival time
21	IL7R	Interleukin 7 receptor	IL7R is overexpressed in adult acute lymphoblastic leukemia and is associatedwith JAK/STAT pathway mutations
22	MME	Membrane metalloendo-peptidase	MME inhibits tumor metastasis of esophageal squamous cell carcinoma and interrupt tumor cell adhesion
23	SKIL	SKI like proto-oncogene	SKIL has regulatory role in cell division or differentiation
24	SMAD7	SMAD family member 7	High expression of SMAD7 at diagnosis predicts poor prognosis in acute myeloid leukemia
25	SMAD1	SMAD family member 1	SMAD1 is involved in a range of biological activities including cell growth, apoptosis, morphogenesis, development and immune responses

## Discussion

MDS is a type of stem cell-derived bone marrow clonal disease with a relatively uneven presentation profile. The incidence of MDS increases with age, the onset of MDS before the age of 40 years is rare. However, when the age is older than 70, the incidence rate exceeds 50 per 100,000 people. In recent years, due to the gradual increase of the population aging problem, the number of patients with MDS has also increased year by year. The exact cause of MDS is uncertain and its relatively risk factors include chemotherapy or radiation therapy and tobacco or poisonous solvents or agricultural chemicals (Tefferi & Vardiman, 2009). In recent years, molecular biology and immune regulation have become increasingly important in MDS (Hosono, 2019; Liu et al., 2019). MDS, also known as pre-leukemia, is characterized by a high risk of transformation to AML. This may be one of the causes of poor prognosis in MDS, so identifying potential markers for MDS diagnosis and prognosis is extremely urgent.

Recently, gene chips have been widely used in research and the corresponding data have been stored in public databases. A number of studies have been conducted based on microarray data profiles to elucidate the mechanisms of disease. But if there is only one data, errors may occur. Therefore, combining bioinformatics methods with expression profiling techniques can partially reduce bias. In this study, I analyzed the expression of genes in two mRNA microarray datasets based on CD34+ cells of bone marrow samples from MDS patients and healthy controls. A total of 133 DEGs were identified, including 15 up-regulated genes and 103 down-regulated genes, however, 15 DEGs were not found in STRING. GO and KEGG pathway enrichment analysis was performed by DAVID online software to explore the biological functions of DEGs. GO enrichment analysis indicated that the identified DEGs were mainly enriched in the immune process. Cancer cells involved in dynamic interference of immune response, evading immune destruction is one of the main features of cancer, involving the participation of a variety of molecular events (Grivennikov, Greten & Karin, 2010; Hanahan & Weinberg, 2011). In a mouse model, IFN-γ-induced apoptosis gene expression promotes destruction of HSCs and progenitor cells (Chen et al., 2015). Interferons are proteins secreted by human immune cells in response to infections (mainly viral). Most of the genes I have identified are immune genes and/or related to the interferon pathway. Therefore, I speculate that the pathogenesis of MDS may be related to immune factors, similar to the study by Silzle et al. (2019) which showed that lymphopenia has a significantly reduced OS in low-risk MDS patients, these data subtly support the role of the immune system in MDS.

In the current study, I selected IFI44L, IRF4, IL7R, SKIL4 as hub genes with the highest degrees. IFI44L, IRF4 and IL7R are immune genes and/or related to the interferon pathway. IFI44L, a type I interferon-stimulated gene, has been shown to be involved in human carcinogenesis and tumor progression (Gao et al., 2013). Pellagatti et al. (2018, 2010) showed that IFI44L was upregulated by more than twofold in at least 60% of the MDS patients and indicated that IFI44L is one of the significant survival predictors in MDS. A recent study found that down-regulation of IRF4 is a necessary and independent effector of immunomodulatory drugs in primary effusion lymphoma (Patil, Manzano & Gottwein, 2018). IRF4 has oncogenic implications in a variety of hematological tumors including multiple myeloma, leukemia and lymphoma (Agnarelli, Chevassut & Mancini, 2018; Cherian et al., 2018; So et al., 2014; Zhang et al., 2013). IL7R is over expressed in adult acute lymphoblastic leukemia (ALL) and is associated with JAK/STAT pathway mutations (Gianfelici et al., 2019). In contrast, IL7R is down-regulated in MDS and this differential expression may be contributed to identifying MDS subtypes and leukemia (Pellagatti et al., 2004). SKIL, also known as SNON, promotes cell invasion of immortalized human mammary epithelial cells. These differential expression could be either cause of the ailment or an effect (or an indirect effect).

The PPI network of DEGs was established and four modules were considered significant in this network. A total of 25 genes were identified in the module models and the functions of these genes were analyzed and Kaplan–Meier curves were employed to predict prognosis. Moreover, the BLNK, IRF4, IFITM1 and ISG20 alteration are significantly associated with worse OS. While the IFIT1 and IFI44L alteration were not statistically significant, they showed an obvious downward tendency. IFI44L and IRF4 have been introduced previously. BLNK (B Cell Linker Protein), which is a selective target of repression, can result in the development of ALL by differentiation block of PAX5-PML protein (Imoto et al., 2016). The results of Nakayama et al. (2009) are consistent with this and somatic cell loss and mutation of BLNK lead to the generation of pre-B-cell leukemia. IFIT1 and IFITM1 are up-regulated in the CD34+ cells of most patients with MDS. IFIT1 and IFITM1 are interferon-stimulated genes (ISG) and up-regulation of IFIT1 is a potential diagnostic marker for MDS (Pellagatti et al., 2006). The ISG20 gene was first introduced by Gongora et al. (1997) as a promyelocytic leukemia nuclear body (PML-NB)-associated protein. The expression of ISG20 is reduced in IDH mutant tumors and ISG20 can promote local tumor immunity and may serve as a potential therapeutic target of human glioma (Gao et al., 2019). This is very interesting: all these identified genes are somehow related to interferons and/or immune response. However, due to a shortfall of the underlying data acquisition technology, false positive results may occur, which is also the biggest flaw of this paper. Next, I plan to collect samples of MDS patients and verify the conclusions of this paper through experiments.

## Conclusion

In conclusion, this study aimed to perform a comprehensive bioinformatics analysis of datasets for MDS patients and healthy controls. Identify DEGs that may be involved in MDS progression and prognosis. Interestingly, most of prognostic genes and hub genes are interferons and immune-related genes. However, further studies are warranted to elucidate the biological function of these genes in MDS.

## Supplemental Information

10.7717/peerj.8162/supp-1 Supplemental Information 1 The gene expression profiles GSE19429 and GSE58831.

## Additional Information and Declarations

### Data Availability

The following information was supplied regarding data availability: The raw measurements are available in the Supplemental File. The gene expression profiles are available at NCBI–GEO: GSE19429, GSE58831.

## References

[ref-1] Adès L, Itzykson R, Fenaux P (2014). Myelodysplastic syndromes. Lancet.

[ref-2] Agnarelli A, Chevassut T, Mancini EJ (2018). IRF4 in multiple myeloma—biology, disease and therapeutic target. Leukemia Research.

[ref-3] Behrens K, Triviai I, Schwieger M, Tekin N, Alawi M, Spohn M, Indenbirken D, Ziegler M, Müller U, Alexander WS, Stocking C (2016). Runx1 downregulates stem cell and megakaryocytic transcription programs that support niche interactions. Blood.

[ref-4] Chen J, Feng X, Desierto MJ, Keyvanfar K, Young NS (2015). IFN-γ-mediated hematopoietic cell destruction in murine models of immune-mediated bone marrow failure. Blood.

[ref-5] Cherian MA, Olson S, Sundaramoorthi H, Cates K, Cheng X, Harding J, Martens A, Challen GA, Tyagi M, Ratner L, Rauch D (2018). An activating mutation of interferon regulatory factor 4 (IRF4) in adult T-cell leukemia. Journal of Biological Chemistry.

[ref-6] Duchmann M, Itzykson R (2019). Clinical update on hypomethylating agents. International Journal of Hematology.

[ref-7] Gao M, Lin Y, Liu X, Li Y, Zhang C, Wang Z, Wang Z, Wang Y, Guo Z (2019). ISG20 promotes local tumor immunity and contributes to poor survival in human glioma. OncoImmunology.

[ref-8] Gao F, Zhao ZL, Zhao WT, Fan QR, Wang SC, Li J, Zhang YQ, Shi JW, Lin XL, Yang S, Xie RY, Liu W, Zhang TT, Sun YL, Xu K, Yao KT, Xiao D (2013). miR-9 modulates the expression of interferon-regulated genes and MHC class I molecules in human nasopharyngeal carcinoma cells. Biochemical and Biophysical Research Communications.

[ref-9] Gianfelici V, Messina M, Paoloni F, Peragine N, Lauretti A, Fedullo AL, Di Giacomo F, Vignetti M, Vitale A, Guarini A, Chiaretti S, Foà R (2019). *IL7R* overexpression in adult acute lymphoblastic leukemia is associated to JAK/STAT pathway mutations and identifies patients who could benefit from targeted therapies. Leukemia & Lymphoma.

[ref-10] Gongora C, David G, Pintard L, Tissot C, Hua TD, Dejean A, Mechti N (1997). Molecular cloning of a new interferon-induced PML nuclear body-associated protein. Journal of Biological Chemistry.

[ref-11] Grivennikov SI, Greten FR, Karin M (2010). Immunity, inflammation, and cancer. Cell.

[ref-12] Hanahan D, Weinberg RA (2011). Hallmarks of cancer: the next generation. Cell.

[ref-13] Hosono N (2019). Genetic abnormalities and pathophysiology of MDS. International Journal of Clinical Oncology.

[ref-14] Imoto N, Hayakawa F, Kurahashi S, Morishita T, Kojima Y, Yasuda T, Sugimoto K, Tsuzuki S, Naoe T, Kiyoi H (2016). B cell linker protein (BLNK) is a selective target of repression by PAX5-PML protein in the differentiation block that leads to the development of acute lymphoblastic leukemia. Journal of Biological Chemistry.

[ref-15] Leite C, Delmonico L, Alves G, Gomes RJ, Martino MR, Da Silva AR, Moreira ADS, Maioli MC, Scherrer LR, Bastos EF, Irineu R, Ornellas MH (2017). Screening of mutations in the additional sex combs like 1, transcriptional regulator, tumor protein p53, and KRAS proto-oncogene, GTPase/NRAS proto-oncogene, GTPase genes of patients with myelodysplastic syndrome. Biomedical Reports.

[ref-16] Liu Y, Bewersdorf JP, Stahl M, Zeidan AM (2019). Immunotherapy in acute myeloid leukemia and myelodysplastic syndromes: the dawn of a new era?. Blood Reviews.

[ref-17] Maciejewski JP, Balasubramanian SK (2017). Clinical implications of somatic mutations in aplastic anemia and myelodysplastic syndrome in genomic age. Hematology.

[ref-18] Makishima H (2019). Genomic aberrations in myelodysplastic syndromes and related disorders. Rinsho Ketsueki.

[ref-19] Mangaonkar AA, Gangat N, Al-Kali A, Elliott MA, Begna KH, Hanson CA, Ketterling RP, Wolanskyj-Spinner AP, Hogan WJ, Litzow MR, Patnaik MM (2018). Prognostic impact of ASXL1 mutations in patients with myelodysplastic syndromes and multilineage dysplasia with or without ring sideroblasts. Leukemia Research.

[ref-20] McReynolds LJ, Zhang Y, Yang Y, Tang J, Mule M, Hsu AP, Townsley DM, West RR, Zhu J, Hickstein DD, Holland SM, Calvo KR, Hourigan CS (2019). Rapid progression to AML in a patient with germline *GATA2* mutation and acquired *NRAS* Q61K mutation. Leukemia Research Reports.

[ref-21] Nakayama J, Yamamoto M, Hayashi K, Satoh H, Bundo K, Kubo M, Goitsuka R, Farrar MA, Kitamura D (2009). BLNK suppresses pre-B-cell leukemogenesis through inhibition of JAK3. Blood.

[ref-22] Ogawa S (2019). Genetics of MDS. Blood.

[ref-23] Patil A, Manzano M, Gottwein E (2018). CK1α and IRF4 are essential and independent effectors of immunomodulatory drugs in primary effusion lymphoma. Blood.

[ref-24] Pellagatti A, Armstrong RN, Steeples V, Sharma E, Repapi E, Singh S, Sanchi A, Radujkovic A, Horn P, Dolatshad H, Roy S, Broxholme J, Lockstone H, Taylor S, Giagounidis A, Vyas P, Schuh A, Hamblin A, Papaemmanuil E, Killick S, Malcovati L, Hennrich ML, Gavin A-C, Ho AD, Luft T, Hellström-Lindberg E, Cazzola M, Smith CWJ, Smith S, Boultwood J (2018). Impact of spliceosome mutations on RNA splicing in myelodysplasia: dysregulated genes/pathways and clinical associations. Blood.

[ref-25] Pellagatti A, Cazzola M, Giagounidis AA, Malcovati L, Porta MG, Killick S, Campbell LJ, Wang L, Langford CF, Fidler C, Oscier D, Aul C, Wainscoat JS, Boultwood J (2006). Gene expression profiles of CD34+ cells in myelodysplastic syndromes: involvement of interferon-stimulated genes and correlation to FAB subtype and karyotype. Blood.

[ref-26] Pellagatti A, Cazzola M, Giagounidis A, Perry J, Malcovati L, Della Porta MG, Jädersten M, Killick S, Verma A, Norbury CJ, Hellström-Lindberg E, Wainscoat JS, Boultwood J (2010). Deregulated gene expression pathways in myelodysplastic syndrome hematopoietic stem cells. Leukemia.

[ref-27] Pellagatti A, Esoof N, Watkins F, Langford CF, Vetrie D, Campbell LJ, Fidler C, Cavenagh JD, Eagleton H, Gordon P, Woodcock B, Pushkaran B, Kwan M, Wainscoat JS, Boultwood J (2004). Gene expression profiling in the myelodysplastic syndromes using cDNA microarray technology. British Journal of Haematology.

[ref-28] Qu X, Zhang S, Wang S, Wang Y, Li W, Huang Y, Zhao H, Wu X, An C, Guo X, Hale J, Li J, Hillyer CD, Mohandas N, Liu J, Yazdanbakhsh K, Vinchi F, Chen L, Kang Q, An X (2018). TET2 deficiency leads to stem cell factor—dependent clonal expansion of dysfunctional erythroid progenitors. Blood.

[ref-29] Shirahata-Adachi M, Iriyama C, Tomita A, Suzuki Y, Shimada K, Kiyoi H (2017). Altered EZH2 splicing and expression is associated with impaired histone H3 lysine 27 tri-Methylation in myelodysplastic syndrome. Leukemia Research.

[ref-30] Silzle T, Blum S, Schuler E, Kaivers J, Rudelius M, Hildebrandt B, Gattermann N, Haas R, Germing U (2019). Lymphopenia at diagnosis is highly prevalent in myelodysplastic syndromes and has an independent negative prognostic value in IPSS-R-low-risk patients. Blood Cancer Journal.

[ref-31] So AY-L, Sookram R, Chaudhuri AA, Minisandram A, Cheng D, Xie C, Lim EL, Flores YG, Jiang S, Kim JT, Keown C, Ramakrishnan P, Baltimore D (2014). Dual mechanisms by which miR-125b represses IRF4 to induce myeloid and B-cell leukemias. Blood.

[ref-32] Tefferi A, Vardiman JW (2009). Myelodysplastic syndromes. New England Journal of Medicine.

[ref-33] Vogelstein B, Papadopoulos N, Velculescu VE, Zhou S, Diaz LA, Kinzler KW (2013). Cancer genome landscapes. Science.

[ref-34] Wang N, Wang F, Shan N, Sui X, Xu H (2017). IDH1 mutation is an independent inferior prognostic indicator for patients with myelodysplastic syndromes. Acta Haematologica.

[ref-35] Weinberg OK, Hasserjian RP (2019). The current approach to the diagnosis of myelodysplastic syndromes. Seminars in Hematology.

[ref-36] Zhang S, Xu J, Wu S, Wang R, Qu X, Yu W, Li J, Chen L (2013). IRF4 promotes cell proliferation by JNK pathway in multiple myeloma. Medical Oncology.

